# Assessment of the therapeutic role of mesenchymal stromal cells in a mouse model of graft-versus-host disease using cryo-imaging

**DOI:** 10.1038/s41598-023-28478-3

**Published:** 2023-01-30

**Authors:** Patiwet Wuttisarnwattana, Saada Eid, David L. Wilson, Kenneth R. Cooke

**Affiliations:** 1 Optimization Theory and Applications for Engineering Systems Research Group, Department of Computer Engineering, Excellence Center in Infrastructure Technology and Transportation Engineering, Biomedical Engineering Institute, Chiang Mai University, Chiang Mai, Thailand; 2 Department of Pediatrics, Case Western Reserve University, Cleveland, OH USA; 3 Department of Biomedical Engineering, Case Western Reserve University, Cleveland, OH USA; 4 Department of Oncology, The Sidney Kimmel Comprehensive Cancer Center at Johns Hopkins Hospital, Johns Hopkins University, Baltimore, MD USA

**Keywords:** Biomedical engineering, Fluorescence imaging, Molecular imaging, Graft-versus-host disease, Immunoproliferative disorders, Bone marrow transplantation, T cells, Stem-cell research, Scientific data

## Abstract

Insights regarding the biodistribution and homing of mesenchymal stromal cells (MSCs), as well as their interaction with alloreactive T-cells are critical for understanding how MSCs can regulate graft-versus-host disease (GVHD) following allogeneic (allo) bone marrow transplantation (BMT). We developed novel assays based on 3D, microscopic, cryo-imaging of whole-mouse-sized volumes to assess the therapeutic potential of human MSCs using an established mouse GVHD model. Following infusion, we quantitatively tracked fluorescently labeled, donor-derived, T-cells and third party MSCs in BMT recipients using multispectral cryo-imaging. Specific MSC homing sites were identified in the marginal zones in the spleen and the lymph nodes, where we believe MSC immunomodulation takes place. The number of MSCs found in spleen of the allo BMT recipients was about 200% more than that observed in the syngeneic group. To more carefully define the effects MSCs had on T cell activation and expansion, we developed novel T-cell proliferation assays including secondary lymphoid organ (SLO) enlargement and Carboxyfluoescein succinimidyl ester (CFSE) dilution. As anticipated, significant SLO volume enlargement and CFSE dilution was observed in allo but not syn BMT recipients due to rapid proliferation and expansion of labeled T-cells. MSC treatment markedly attenuated CFSE dilution and volume enlargement of SLO. These assays confirm evidence of potent, in vivo, immunomodulatory properties of MSC following allo BMT. Our innovative platform includes novel methods for tracking cells of interest as well as assessing therapeutic function of MSCs during GVHD induction. Our results support the use of MSCs treatment or prevention of GVHD and illuminate the wider adoption of MSCs as a standard medicinal cell therapy.

## Introduction

Allogeneic (allo) bone marrow transplantation (BMT) remains the only curative therapy for a number of malignant and non-malignant conditions^1,2^. Unfortunately, graft-versus-host disease (GVHD) continues to limit successful outcomes even though our understanding of the biology of this disorder has evolved considerably over the last several decades^3,4^. The disease-inducing cells are known to incude donor-derived T-cells contained within the donor bone marrow graft^5,6^. GVHD target organs include the lung, liver, skin and intestinal tract^6–8^. The pathophysiology of acute GVHD is complex but can be conceptualized in distinct phases^4,9^. Diffuse, non-specific, damage to host tissues from BMT conditioning regimens sets the stage for activation of donor T-cells infused in the bone marrow inoculum. In the final stage, GVHD target organs are damaged by the activated T-cells and soluble inflammatory proteins like TNFα and IL-6^3,4,10^. While simplistic, this three phase hypothesis underscores opportunities for new therapies for GVHD to be developed and tested^11^.

The traditional approach to prevent or treat GVHD is to use immunosuppressive agents that disrupt T-cell activation and proliferation^12^. Steroids are the first line of treatment for acute GVHD^1,2^, but only half of patients respond to steroids and those with steroid-refractory acute GVHD have a dismal outcome^1–3,13–15^. Furthermore, disruption of immune responses increases the risk of opportunistic infections as well as the chance of relapse of underlying malignancy. The development of novel therapies to effectively treat or prevent GVHD remains a significant knowledge gap in the field.

In this context, mesenchymal stromal cells (MSCs) have been applied to the treatment of GVHD. MSCs are multipotent stromal cells capable of self-renewal and differentiation into various types of tissues^16^. They have gained the attention of the scientific community for their use in cellular therapy and regenerative medicine. The potential utility of MSCs is not limited to their regenerative capacity; MSCs have potent immunomodulatory effects, and their ability to secrete growth factors that regulate local immune cells and inflammation in the surrounding microenvironment likely contributes to their clinical utility^17^. Evidence for this has been demonstrated in vitro^18,19^ and in a number of in vivo models^20,21^. MSCs are currently being studied in several clinical trials for preventing immune-mediated tissue damage and facilitating repair after injury^22^. However, the mechanisms responsible for these immune modulating effects along with the bio-distribution of MSCs after infusion in both pre-clinical and clinical studies remain elusive. Not all clinical trials have been successful^23^, and the scientific consensus is that timing, dose and schedule are all critical elements to optimize therapeutic efficacy^24^. Optimal timing relates in part to the necessity for MSCs to be activated or “primed” before exerting their immunomodulatory effects by proteins including, but not limited to, IFNγ in combination with IL-1, IL-6, and TNFα^25,26^ and prostaglandin E2 (PGE2)^27–29^. Moreover, some reports suggest that MSC-mediated immunosuppression requires cellular contact between MSCs and the effector T-cells^30^, and others have demonstrated that MSCs home to secondary lymphoid organs (SLO) where T-cell activation and proliferation take place^31^. Hence the details of MSC bio-distribution, homing, and interaction with T-cells in SLOs are critical for understanding their role in GVHD therapy, but remain incompletely characterized and understood. Furthermore, limitations of traditional imaging technologies and the development of suitable models to study human MSCs contribute to this knowledge gap.

Imaging has been widely used to visually assess and quantitatively measure lymphocyte function. Bioluminescent imaging (BLI) is used as an assay to study cellular engraftment and proliferation in animal models^32^. Two-photon and confocal microscopy have been employed to study real-time function of lymphocytes^33^. Non-invasive positron emission tomography (PET) imaging can assess metabolic activity in vivo at specific cross sections in time and has significant utility in the staging of cancer and monitoring of tumor response to therapy^34^. However, none of these techniques provide whole body imaging with single cell sensitivity which is crucial for systematic assessment of cellular function and precise bio-distribution. To address this unmet need, we developed a novel lymphocyte proliferation assessment protocol based on cryo-imaging. Cryo-imaging^35–38^ is a fully-automated, whole mouse, section-and-image system, which provides 3D, tiled, microscopic, anatomical brightfield and molecular fluorescent images over vast volumes. It provides single cell detection anywhere in the mouse and determines cell densities far below what can be observed with any other imaging technologies such as MRI, CT, PET, SPECT and BLI. Our protocol includes two parameters: SLO volume enlargement and carboxyfluorescein succinimidyl ester (CFSE) dilution.

The goal of our paper is to determine the location and degree of MSC immunomodulation of GVHD using an established pre-clinical system. We hypothesize that early MSC trafficking to SLOs and co-localization with T-cells are the key events that lead to the therapeutic effects of MSCs. We applied recently described methods (whole mouse microscopic multi-spectral cryo-imaging, mouse tissue segmentation algorithms^39–41^, machine-learning stem cell analysis software^42^) and new quantitative approaches to spatially assess in vivo, T-cell proliferation in cryo-images using CFSE-dilution in order to test this hypothesis.

## Methods

### Mouse model of graft-versus-host disease

BMT was performed as previously described^21,43,44^. Female C57BL/6 J (B6) and B6D2F1 (F1) mice aged 8 to 12 weeks were purchased from Jackson Laboratory (Bar Harbor, ME). Prior to BMT, lethal total body irradiation (14 Gy) was given as a split dose to all F1 recipients^21,44^. Bone marrow (5 million) and T-cells (2 million), collected from either allo B6 or Syn F1 donor mice, were suspended in 200 µl Leibovitz L-15 media and intravenously injected into F1 recipient mice on day 0 (T = 0). T-cell purification was performed by magnetic-bead separation using MicroBeads and the autoMACS system (Miltenyi Biotec, Auburn, CA) with more than 85% of cells obtained being positive for the CD3 surface antigen. T-cells were fluorescently labeled with CFDA-SE dyes (Vybrant^®^ CellTracer, Life Technologies Corp.) prior to injection. In this model, allo BMT recipients developed reproducible acute GVHD as assessed by survival, clinical score, target organ histopathology and donor T cell activation and expansion, while the recipients of syn BMT did not^21,45^. IACUC and the Case Animal Resource Center (ARC) approved the mouse protocol used in this paper (IACUC protocol number 2010-0076). We confirm that all experiments were performed in accordance with relevant guidelines and regulations. We also confirm that our study is reported in accordance with ARRIVE guidelines.

MSCs were used as a strategy to reduce GVHD^21^. Human MSCs were derived from BM aspirates from healthy donors collected and processed by the Hematopoietic Stem Cell Facility of the Case Comprehensive Cancer Center. MSC collection and processing are described in the supplementary data. 1 × 10^6^ human MSCs were labeled with red quantum dots (QTracker™ 625, Life Technologies). The cells were then injected into allo and syn BMT mice on Day +1 or 24 h after BMT. As controls, 1 × 10^6^ unlabeled MSCs were injected into some allo and syn BMT recipients. We allowed sufficient time (24 h post injection) for the MSCs to circulate and home naturally. Animal were killed (anesthetization by isoflurane and euthanized by carbon dioxide) for analysis at specific time points (24, 48, 72, and 96 h) after BMT. To prepare for cryo-imaging, whole animals were embedded in OCT medium (Tissue-Tek, Sakura Fintek USA Inc.) inside a custom freezing apparatus, snap frozen in liquid nitrogen, and mounted on to the cryo-imaging specimen stage.

### Cryo-imaging and analysis software

The whole-mouse 3D microscopic cryo-imaging system (CryoViz™, BioInVision Inc.) consists of a fully automated, section-and-image system, which includes a whole-mouse cryo-microtome, microscope imaging system, robotic positioner, and specialized visualization/analysis software. CryoViz™ provides 3D, tiled, microscopic, anatomical bright field and molecular fluorescent volumes over an entire mouse^36^. CryoViz™ permits unique quantitative analyses of fluorescently-labeled, single cells using specialized image analysis and visualization software^37,38,46^. The samples were sectioned at 40 μm in the CryoViz™ at a temperature of -20ºC. Magnification of the microscope was set so that the pixel size was 10.5 μm. With our standard imaging configuration, the voxel size is set to be sufficiently small (about the size of a single cell) to avoid the partial volume effect. Amira (Thermo Fisher Scientific, MA) and Matlab (MathWorks Inc., MA) software were used for 3D visualization and algorithm development, respectively.

### MSC and T-cell detection algorithms

Red-fluorescent MSCs and green-fluorescent T-cells were detected and quantified anywhere in the whole-mouse volume. We previously developed a specialized, machine learning, algorithm for detecting red fluorescently labeled MSCs^35,42^ In brief, we extracted 4 features derived from the fluorescent images including top-hat transforms of red and green channels as well as the Mexican hat filtering of the two channels. We then employed bagged decision trees machine learning algorithm to recognize if the pixels, in the form of the feature vectors, are the signal from MSCs. The detected signals were grouped together using 3D connected component analysis to prevent double counting that could be a result from subsurface fluorescence. The algorithm has been shown to robustly detect most of the MSC signals while rejecting the auto-fluorescent signals and noise^42^. In this work, we specifically quantified cells in particular organs (e.g., lung, liver, spleen, kidneys, bone marrows, lymph nodes, brain, and other tissues).

A T-cell detection algorithm was also developed^46^. To detect T-cells, the green fluorescent channel was filtered using a Mexican hat kernel, which is also known as a flipped Laplacian of Gaussian (LoG). The parameter sigma (σ) for the LoG was chosen such that the kernel was well-matched to the shape of the cells. Then, a threshold (*T*_*isolated*_) was applied to the filtered image to mark location of T-cells. The threshold was empirically adjusted such that T-cells were mostly marked. The same threshold was also tested with control images containing only the tissue autofluorescence with unlabeled T-cells. This threshold would minimally mark the autofluorescent pixels. With the aid of 3D visualization tool, we manually removed any detected signals that deemed to be false detections outside the plausible tissues. For detecting larger clusters of cells, the kernel size (in term of σ) were increased and adjusted, and then the whole process was repeated to include T-cells in different size. To quantify a single cell intensity, top-hat transform was applied to the fluorescent image to remove the autofluorescent background from the detected signals.

### SLO volume enlargement assay

SLO volumes were used to assess T-cell proliferation during the development of GVHD. The SLO enlargement assay is predicated on the expectation that the volume of SLOs (spleen and lymph nodes) will increase due to the rapid clonal expansion of lymphocytes^47–49^. Hence, we hypothesize that the volume of the SLOs following allogeneic BMT group will be significantly larger than that seen in control animals with no GVHD.

SLOs enlargement assays were principally developed based on spleen and lymph nodes tissues. In spleen, the white pulp region is a lymphocyte-rich compartment, where donor-derived, alloreactive, T-cells will home and proliferate^50^. For lymph nodes, we expect that exogenous T-cells will specifically home and proliferate in the paracortex region of lymph node^51^. White pulp volume (mm^3^), total spleen volume (mm^3^), and the percentage of white pulp volume to total spleen volume (%WP) were used as three metrics for assessing spleen enlargement. Since the spleen image data were quite large, we have developed a specialized algorithm to automatically label red pulp and white pulp regions in the spleen images. The software development is described in detail elsewhere^39,40^. For lymph node analysis, we chose inguinal lymph nodes (iLN) and cervical lymph nodes (cLN) for assessment. Whole lymph node volume was used as the metric of lymph node size. Because lymph nodes were much smaller in size, we utilized a 3D segmentation tool to manually delineate the lymph node area in the cryo-images. Since T-cells were labeled with fluorescent dyes, the T-cell rich zone was highly fluorescent, a cue that aided the segmentation. The volume of any tissue of interest was calculated by multiplying the number of segmented voxels with the voxel size (mm^3^).

### CFSE dilution assay

CFSE intensity was used to assess T-cell proliferation in the SLO. The CFSE dilution assay is an accepted measure of T cell proliferation using flow cytometric-based measurements^52,53^. By labeling T-cells with a fixed number of fluorescent molecules, one can assess proliferation from the diminution (i.e. dilution) of signal as the cells divide separating fluorescent molecules into daughter cells^54^. Since CFSE dyes are covalently bound to the intracellular molecules, CFSE dye levels in non-dividing cells remain relatively stable^54^. Over time, the highly proliferative cells will fluoresce less brightly while non-proliferating T-cells maintain high level of CFSE concentration. We have applied this knowledge to design our cryo-imaging-based CFSE dilution assay^49^. Our CFSE dilution assays included color-coded volume rendering of cell intensity and quantitative evaluation of the CFSE cell intensity histogram. Volume rendering of CFSE intensity allows qualitative assessment of T-cell proliferation. To create the volume rendering, spleens and lymph nodes were manually segmented from the cryo-imaging data. Green channels of the fluorescent images, which contained CFSE information, were extracted to form a 3D volume. The 3D data were rendered using a standard rainbow color map where red color indicates higher CFSE intensity and blue color indicates lower CFSE intensity*.* Fluorescent intensity values of 40 and 130 (on the scale of 255) for defining blue color (min) and red color (max) thresholds were deemed best for representing CFSE signal over the background signal in spleens. For lymph node visualization, we used the range of 60 and 200 to represents min and max of the color map. We hypothesized that the allo volume visualization would shift the color from red to blue faster than the syn control as the T-cells lost their fluorescence during the rapid clonal expansion in the disease group.

We analyzed the T-cell intensity histograms in the form of probability density function (PDF) to quantitatively assess T-cell proliferation. To analyze the T-cell intensity, we applied the T-cell detection algorithm to the SLO volumes as described in the previous section. A list for each T-cell intensity assessment was obtained by the algorithm. Next, the list was used to estimate the PDF using the Kernel Density Estimation technique^55^. We explored different analyses of the PDFs, but settled on what we call “%High.” A single threshold (*T*_ref_) for all data was empirically chosen to partition high and low intensity cells. The %High value was given by the area under the curve (AUC) of high intensity voxels in the PDF. We expected that %High of the syngeneic group would be significantly higher than that of the allogeneic group, indicating minimal T-cell proliferation in the syngeneic group, and vice versa. This would prove the validity of our assay.

For all statistically analyses in this work, we used two-tailed Student’s t-test to evaluate metrics between the study group and the control group (* and ** represent *p*-value < 0.05 and 0.005 respectively).

## Results

### Co-localization of T-cells and MSCs in the secondary lymphoid organs

**Figure 1:**
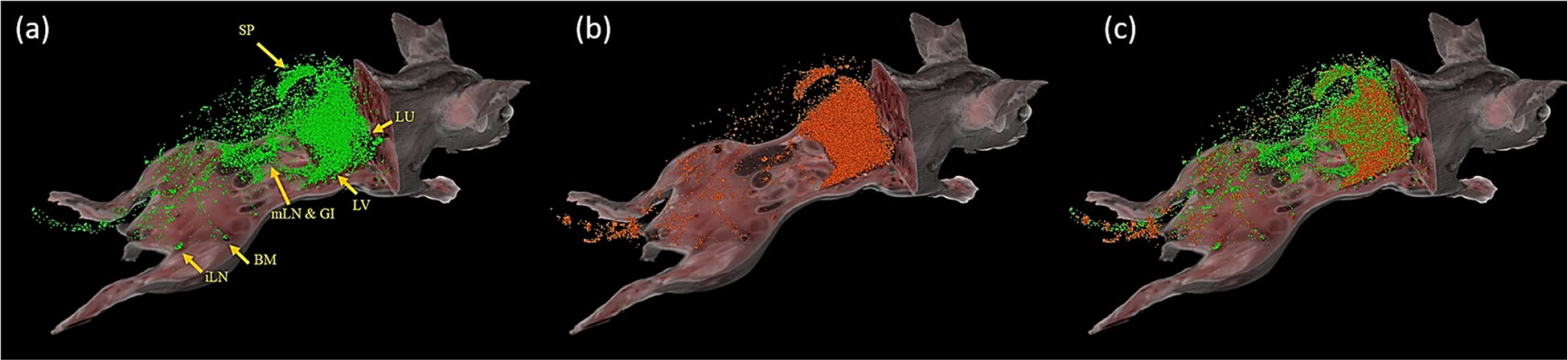
Multispectral cryo-imaging provides co-localization of multiple cell types. Cryo-imaging has enabled us to obtain biodistribution of green CFSE-labeled T-cells (**a**), red Qdot-labeled MSC’s (**b**) as well as co-localization of both cell types (**c**). Allogeneic bone marrow transplantation was performed wherein allogeneic T-cells (2 million) and bone marrow cells were infused on day 0, and MSCs (1 million) were infused on day 1. Mouse sacrifice was performed on day 2, followed by cryo-imaging. Abbreviations in (A): SP: Spleen, LU: Lung, LV: Liver, mLN: mesenteric lymph nodes, GI: Gastrointestinal tissues, BM: Bone marrow, iLN: inguinal lymph nodes. Imaging time = 48 post T-cell injection into allogeneic BMT receipients. See also provided movie in supplemental media 1. Note that Fig. 1a was reproduced from Wuttisarnwattana et al.^49^ with permission from SPIE.

Multi-spectral cryo-imaging enables T-cell and MSC tracking in the entire mouse with single cell sensitivity (Fig. 1). Labeled, donor, T-cells were found in lung, liver, bone marrow, GI-tract, and secondary lymphoid organs (Fig. 1a). MSCs were primarily found in lung, liver, spleen, and bone marrow (Fig. 1b). Interestingly, we observed that MSC densities in kidney, heart, muscle, adipose tissue, brain, and spinal cord were very low, regardless of the high cardiac output directed to some of these tissues (Suppl. Media 1). Finally, co-localization of both cell types was also obtained (Fig. 1c). This interactive 3D visualization suggests potential interaction sites of the effector cells where immunomodulation takes place. The video in supplemental media 1 further illustrates cellular biodistribution and co-localization.

**Figure 2:**
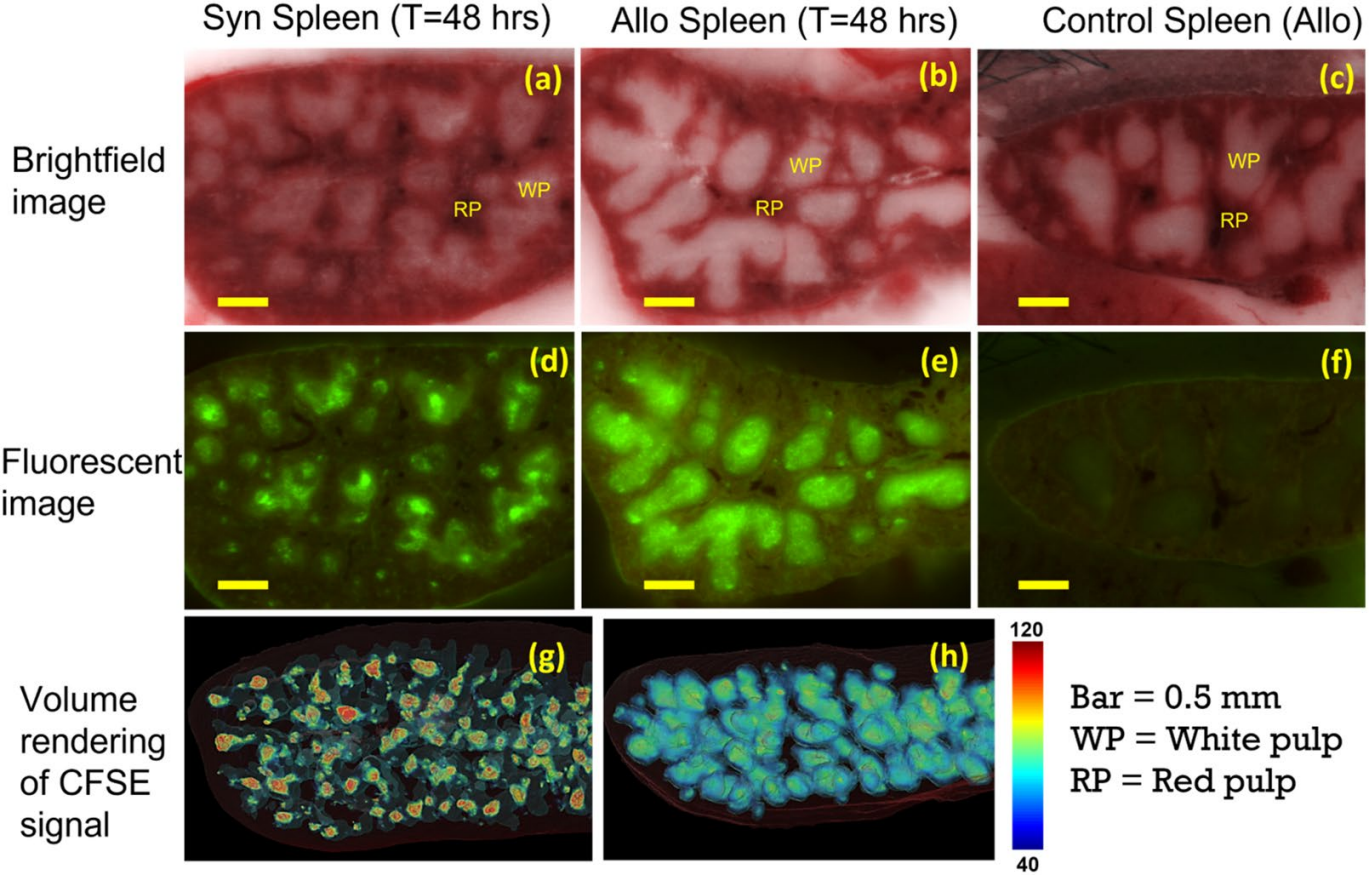
CFSE-labeled T-cells homed specifically to the white pulp of spleen. Brightfield images show that spleen consists of red pulp (RP) and white pulp (WP) (**a**–**c**). Homing sites of exogenous T-cells are clearly visible in fluorescent images (green clusters in **d**, **e**). At 48 h post BMT, allogeneic T-cells occupied the entire white pulp while syngeneic T-cells only occupied the white pulp partially (**d**, **e**). Color-coded volume rendering of CFSE intensity in the spleen also shows that T-cells in the Allo group rapidly proliferated and lost CFSE intensity as compared to the spleen of the Syn group (**g**, **h**). Unlabeled T-cells in the control group did not fluoresce (**f**).

**Figure 3:**
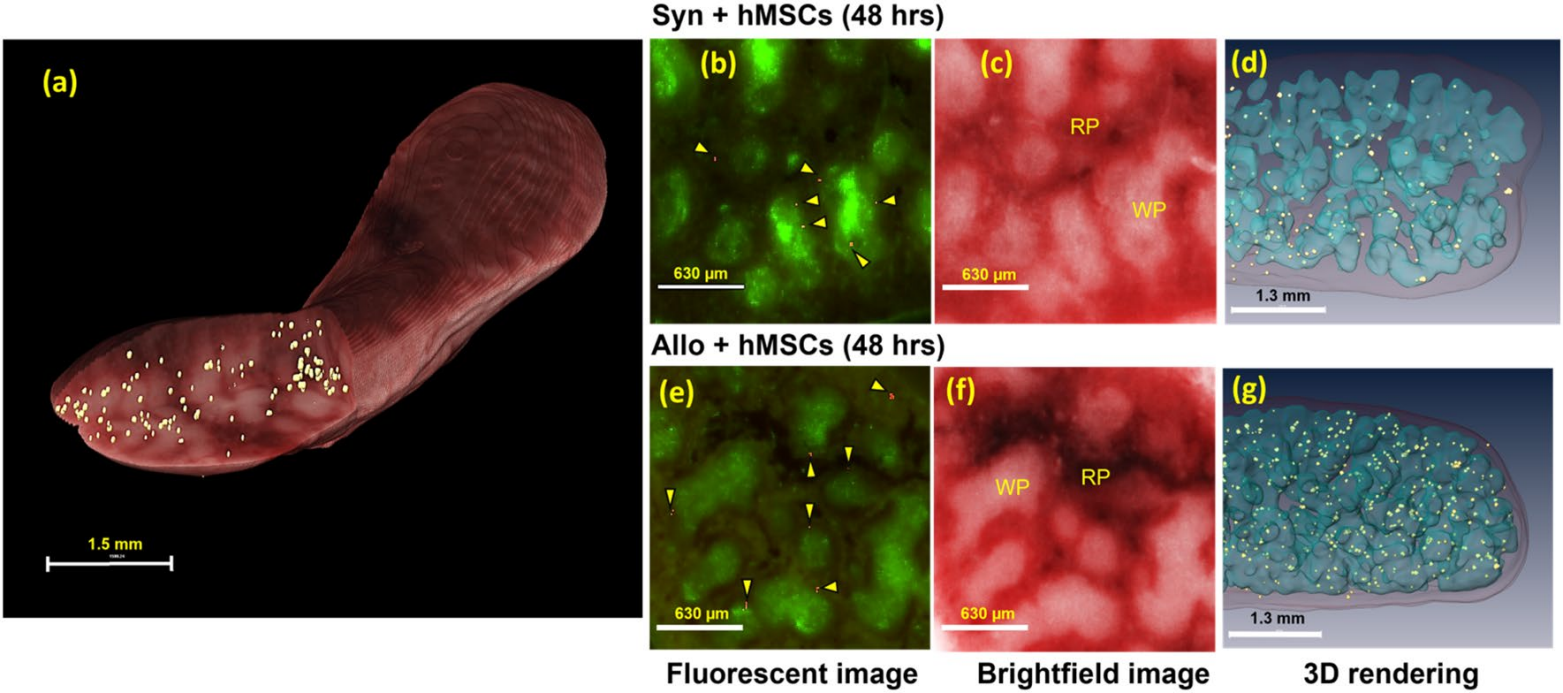
hMSCs were substantially found in the spleen which is the largest secondary lymphoid organ. Detected hMSCs were rendered in golden yellow (pseudo-color) in the spleen volume rendering (**a**). Fluorescent images show that red, Qdot-labeled, MSCs (arrow heads) were found to home specifically to the marginal zone (**b**, **e**) which is the interface between the red pulp (RP) and the white pulp (WP) (Subpanels c, f and movies in supplemental media 2–3). 3D visualizations of white pulp (blue surface renderings in **d** and **g**) show that there were more hMSCs in the spleen of the allogeneic group than in the spleen of the syngeneic group.

Donor T-cells and MSCs co-localized in immuno-active regions of the spleen, and results were accentuated in the presence of GVHD following allo BMT as compared to syn controls. Brightfield microscopic imaging, demonstrates the red pup and white pulp tissues of the spleen (Fig. 2a–c). Fluorescently labeled, donor-derived, T-cells specifically accumulated in the white pulp (as opposed to the red pulp) of the spleen (Fig. 2d,e), consistent with known physiology. Interestingly, at early time points (T = 48 h), allogeneic T-cells occupied the entire white pulp (Fig. 2e,h) while the syngeneic T-cells only partially filled the white pulp (Fig. 2d,g). As anticipated, unlabeled T-cells in the control group did not fluoresce (Fig. 2f). Fluorescently labeled MSCs were also found in the spleen (Fig. 3). They preferentially homed to the marginal zone, which is the interface between white pulp and red pulp (Fig. 3b,e and supplemental media 2–3). Interestingly, we found that the number of MSCs in spleens of allo mice was significantly higher than that found in the spleens of syn mice (Figs. 3d,g and 6a). The numbers of detected MSCs in spleen were listed in Suppl. Table 1. On average, the number of MSCs found in spleen was doubled in the allo group as compared to the number in the syn group.

**Figure 4:**
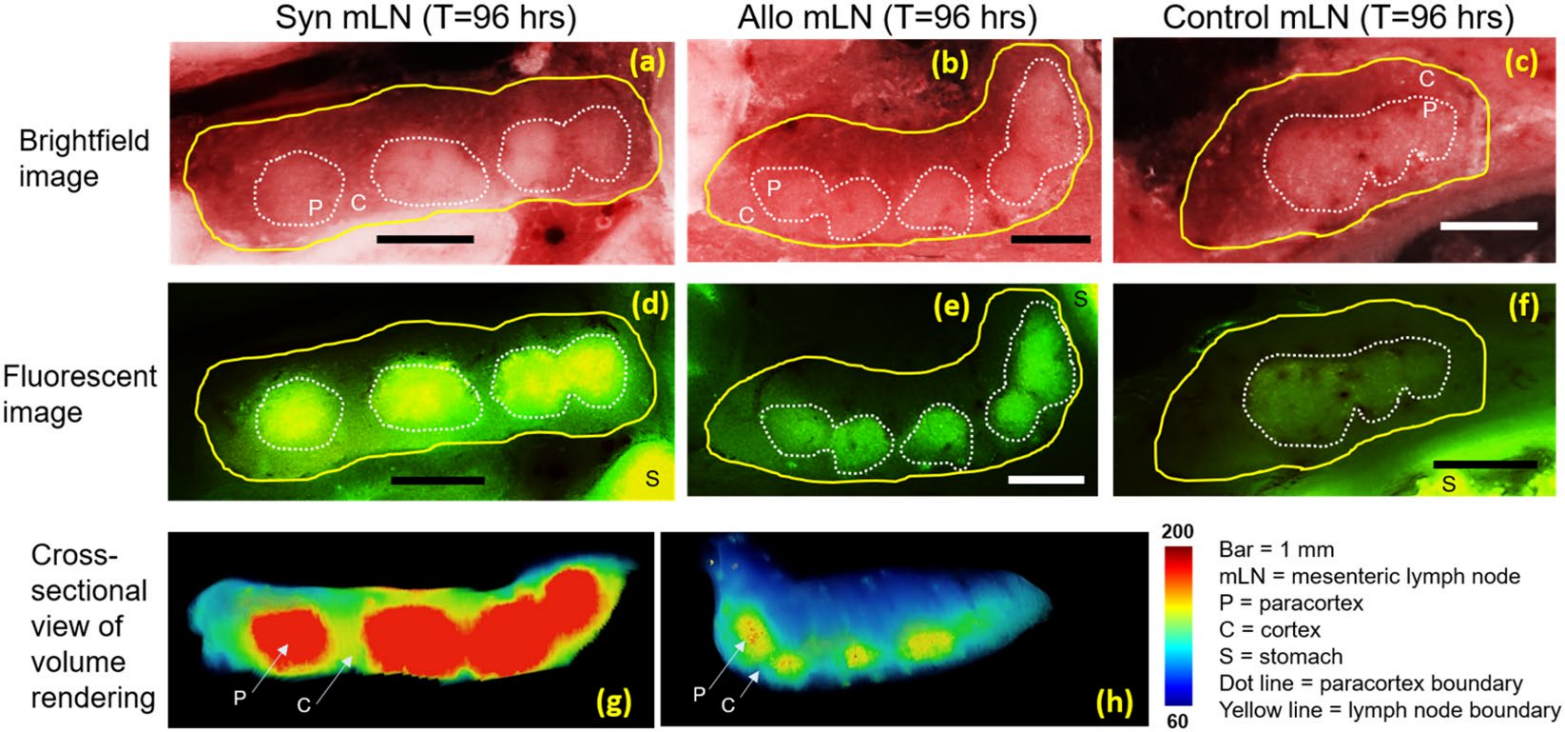
CFSE-labeled T-cells homed specifically to the paracortex of lymph nodes. Brightfield images show that mesenteric lymph node consists of cortex (C) and paracortex (P) substructures (**a**–**c**). In fluorescent images, homing sites in the paracortex regions are visible as green clusters (**d**, **e**). We hypothesize that both types of exogenous T-cells came to the lymph nodes (**d**, **e**) but only the alloreactive T-cells were primed and proliferated (**e**). Color-coded volume rendering of CFSE intensity in lymph node also shows that the allogeneic T-cells significantly lost CFSE intensity as compared to syngeneic T-cells which indicates rapid proliferation (**g**, **h**). Autofluorescence of lymph nodes of the control group was negligibly low (**f**).

**Figure 5:**
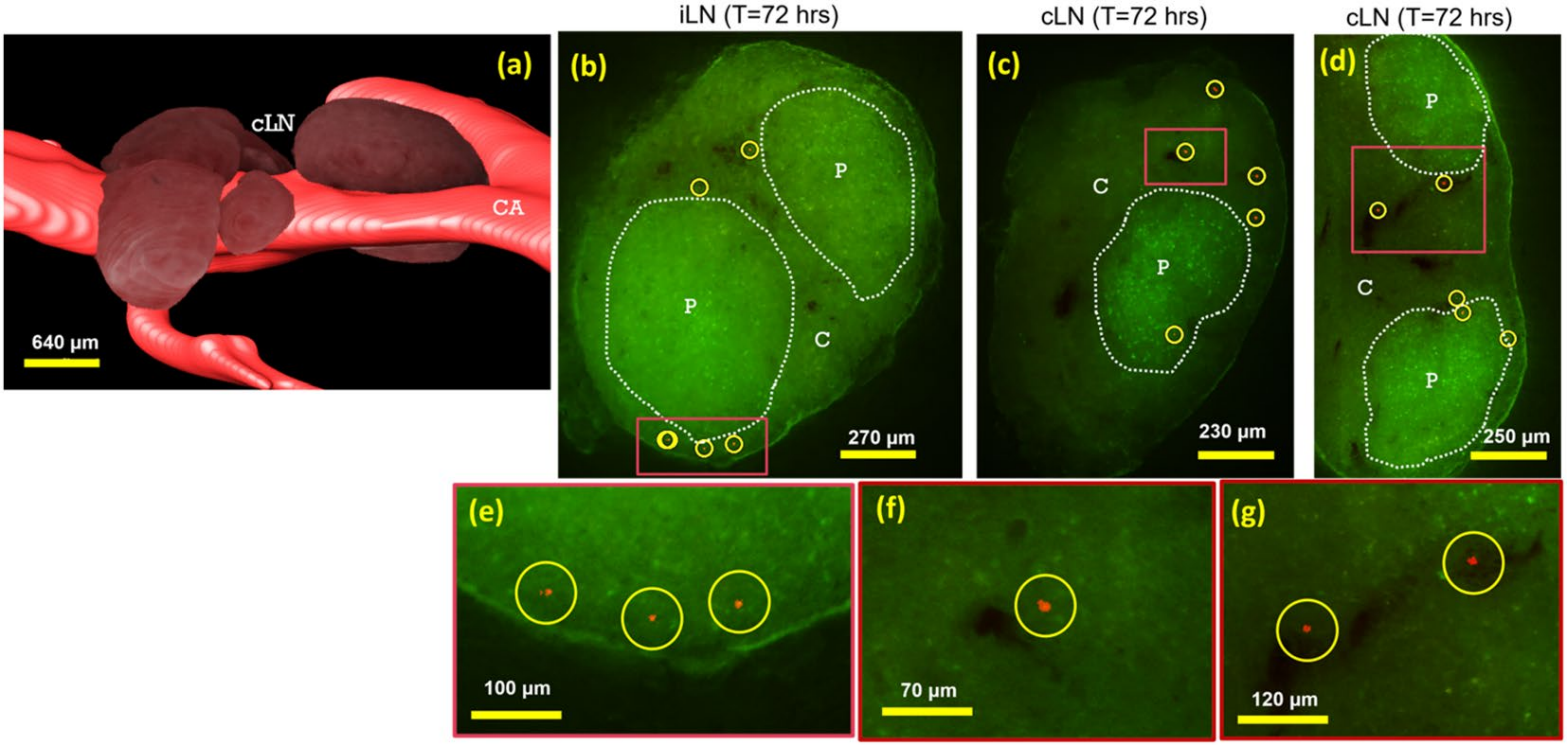
hMSC homing in lymph nodes. Volume rendering (**a**) shows anatomical appearance of murine cervical lymph nodes (cLN) juxtaposed with the carotid artery (CA). Fluorescent images show that red Qdot-labeled hMSCs were found in lymph nodes (**b**–**g**). We manually identified lymph nodes throughout the mouse. These included inguinal lymph nodes (iLN), and cervical lymph nodes (cLN, as in **a**). Interface between the cortex (C) and the paracortex (P) regions of lymph nodes are highlighted with white dotted lines. hMSCs were found just outside the paracortex (Yellow circles and arrow heads). In most cases, they were found in close proximity of the blood vessels (dark regions in lymph nodes). See also movies in the supplemental media 4.

**Figure 6:**
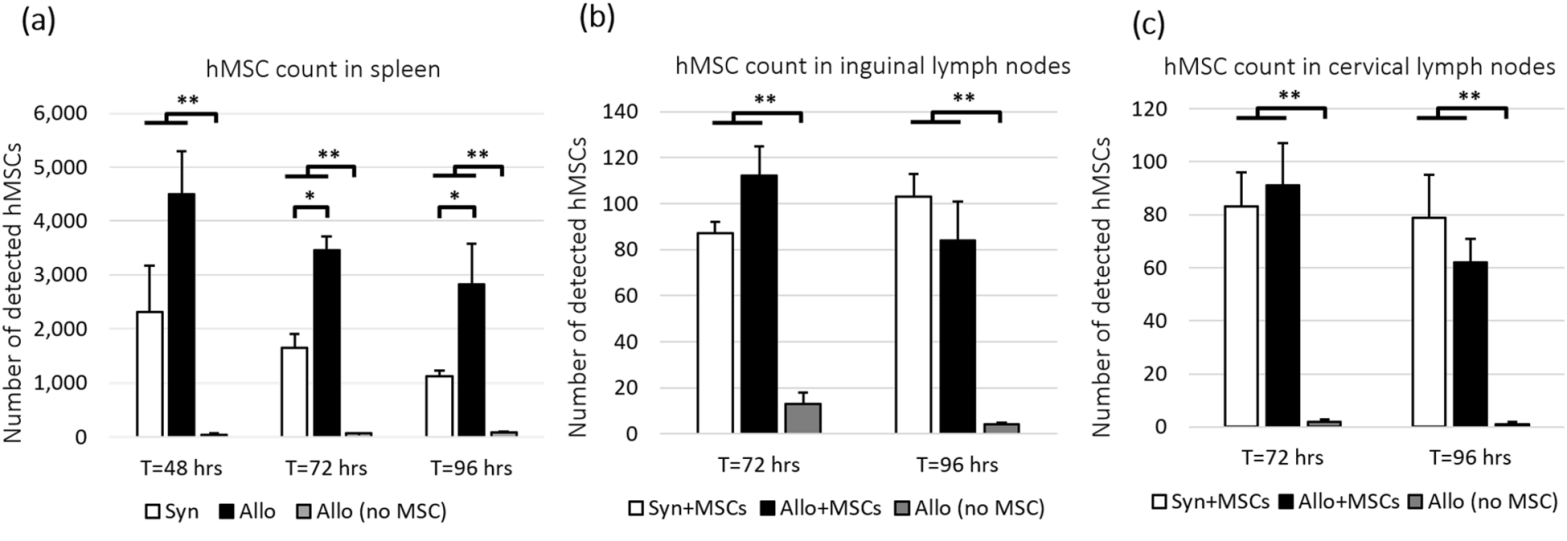
MSCs were found in spleen (**a**) and lymph nodes (**b**, **c**). However, only number of MSCs in spleen of allogeneic mice was significantly higher than that in spleens of syngeneic mice. We speculate that the preferential recruitment of MSCs was a response to the T-cell priming and activation in the spleens of allogeneic mice. We hypothesize that MSCs homing to the spleen was responsible for immunomodulation. False detections in control spleens (injected with unlabeled cells) were negligible. Error bar represents standard error. Annotations * and ** represent significantly difference with *p*-value < 0.05 and 0.005, respectively.

Both exogenous T-cells and MSCs also homed to lymph nodes (Figs. 4 and 5), and results were again accentuated by the development of GVHD. Fluorescent images of mesenteric lymph nodes show that CFSE-labeled T-cells were found to home specifically to paracortex region (Fig. 4d,e). As expected, alloreactive T-cells were observed to proliferate and lose their CFSE signal to a greater extent (Fig. 4e, h) than syngeneic T-cells (Fig. 4d,g). Unlabeled T-cells in the control group had very low auto-fluorescent signal (Fig. 4f). To assess MSC homing in lymph nodes, we specifically analyzed the inguinal and cervical lymph nodes (Fig. 5a) where MSCs could also be found (Fig. 5b–g and supplemental media 4). In most cases, they were just outside the paracortex and in close proximity of the blood vessels. Unlike in spleen, the numbers of detected MSCs in the lymph nodes of allo mice were comparable to syngeneic controls. (Fig. 6b,c). The numbers of detected MSCs in iLN and cLN are listed in Suppl. Table 2.

### Spleen and lymph node volumes

**Figure 7:**
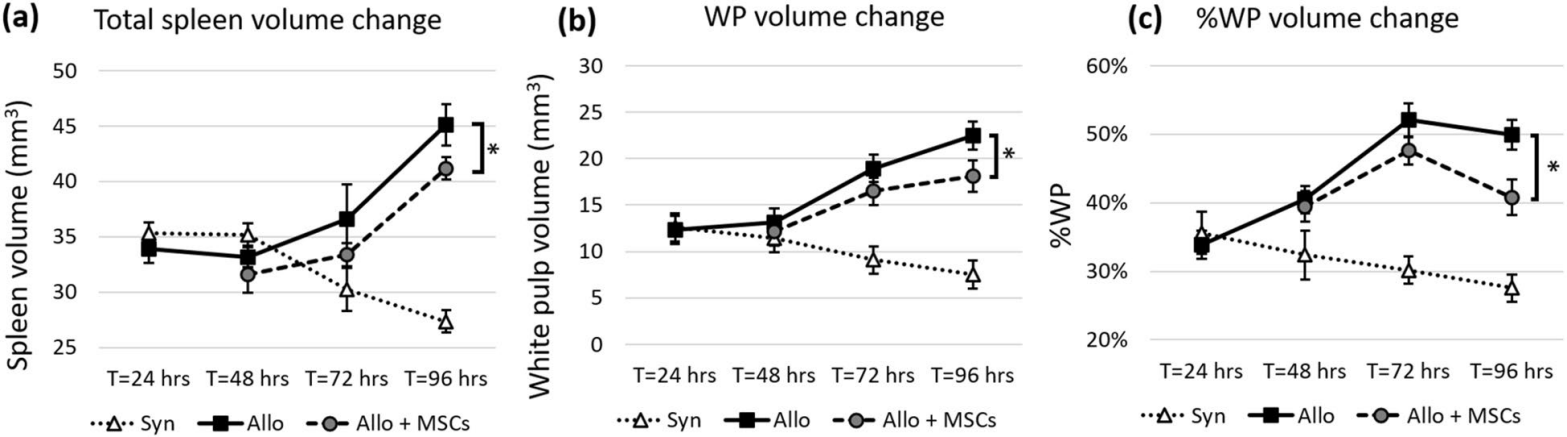
Spleens from allogeneic group greatly increased in size as a result of rapid proliferation. With hMSC treatment, spleen enlargement was significantly attenuated. We proposed three metrics to assess T-cell proliferation in spleen—Total spleen volume (mm^3^), WP volume (mm^3^) and %WP volume to total spleen volume. Metrics show that spleens of the allogeneic group were bigger than that of the syngeneic group. The spleens from the allogeneic group with MSC treatment were significantly smaller than the spleens from the allogeneic group without MSC treatment. This suggests that hMSCs could suppress T-cell proliferation in the allogeneic group. The error bar represents standard error. Annotation * represents significantly difference with *p*-value < 0.05 (Two-tailed Student’s t-test).

**Figure 8:**
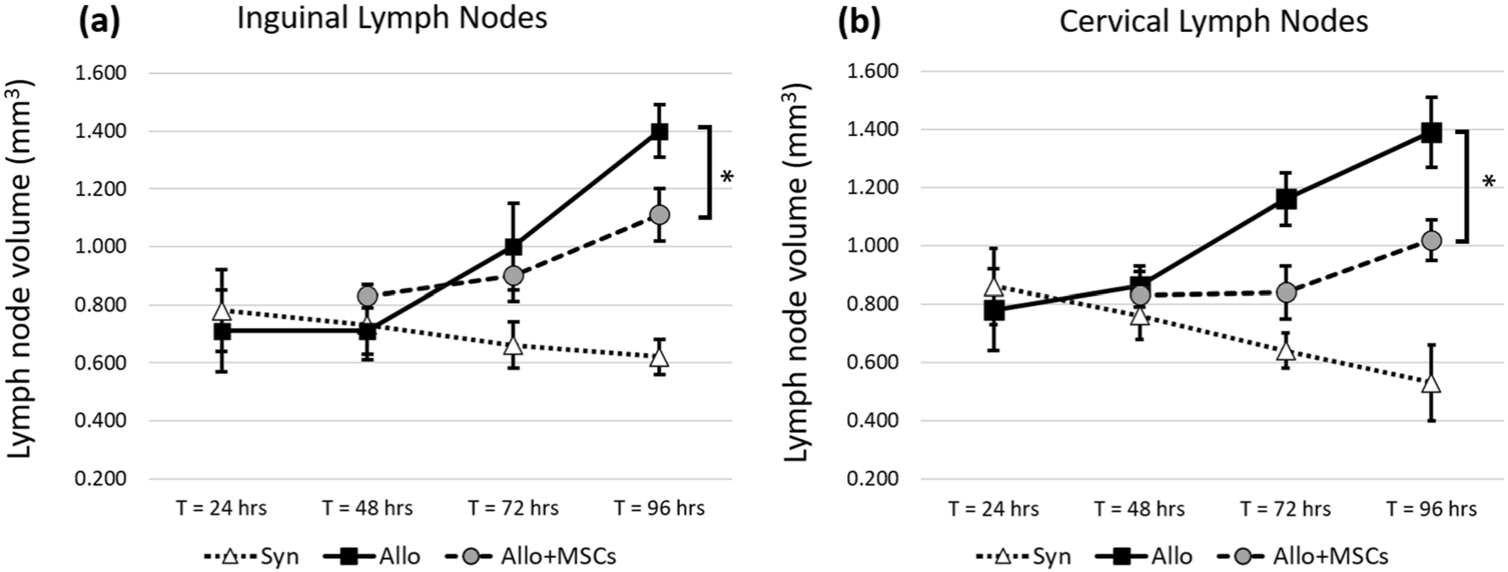
Lymph nodes from mice in the allogeneic group greatly increased in size as a result of rapid proliferation. With hMSC treatment, lymph node enlargement was significantly attenuated. We measured lymph node volume of inguinal and cervical lymph nodes. Results show that lymph nodes from allogeneic group were significantly bigger than the lymph nodes from syngeneic control. The assay suggests that hMSCs could suppress T-cell proliferation in the allogeneic group. The error bar represents standard error. Annotation * represents significantly difference with *p*-value < 0.05 (Two-tailed Student’s t-test).

Coincident with the development of GVHD, SLOs of allo BMT recipients were significantly larger than SLOs of syn mice. The size of spleens from allo mice increased significantly starting at T = 48 h post BMT (Fig. 7a–c) through 96 h post BMT (*p*-value < 0.005). Whole lymph node volumes were also bigger in allo mice compared to syn controls. The enlargement became significant by 72 h post BMT (*p*-value < 0.05 at T = 72 and *p*-value < 0.005 at T = 96 h post BMT). The results were consistently observed within inguinal and cervical lymph nodes (Fig. 8). Volume renderings of the organs were consistent with the quantitative results (Suppl. Figs. 1 and 2). When compared to the allo controls without MSC treatment, SLO enlargement in the MSC treated group was greatly reduced (Fig. 7a–c). The reduction in SLO enlargement became significant by 96 h post BMT (*p*-value < 0.05). Likewise, lymph node enlargement of the allo group was significantly reduced in the MSC-treated group (Fig. 8). We observed that the reduction in enlargement of cervical and inguinal lymph nodes became significant by 96 h post BMT (*p*-value < 0.05).

### CFSE dilution in spleen and lymph nodes

**Figure 9:**
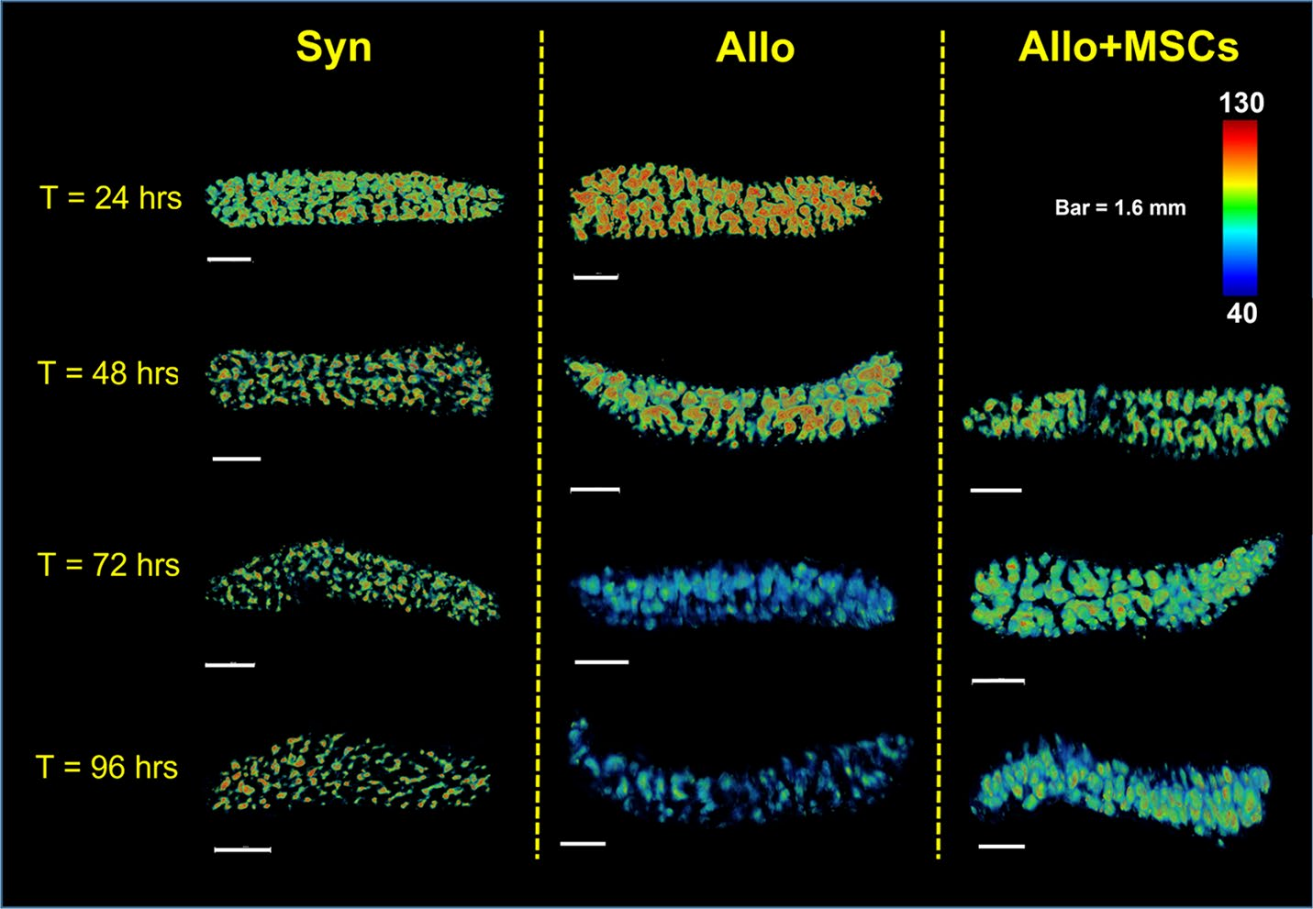
Volume rendering of CFSE signal in the spleen shows that alloreactive T-cells lost their CFSE intensity over time as they proliferated (second column). With MSC treatment, CFSE dilution was attenuated (third column). In the syngeneic control, T-cells in the spleens maintained high CFSE intensity which indicated minimal proliferation.

**Figure 10:**
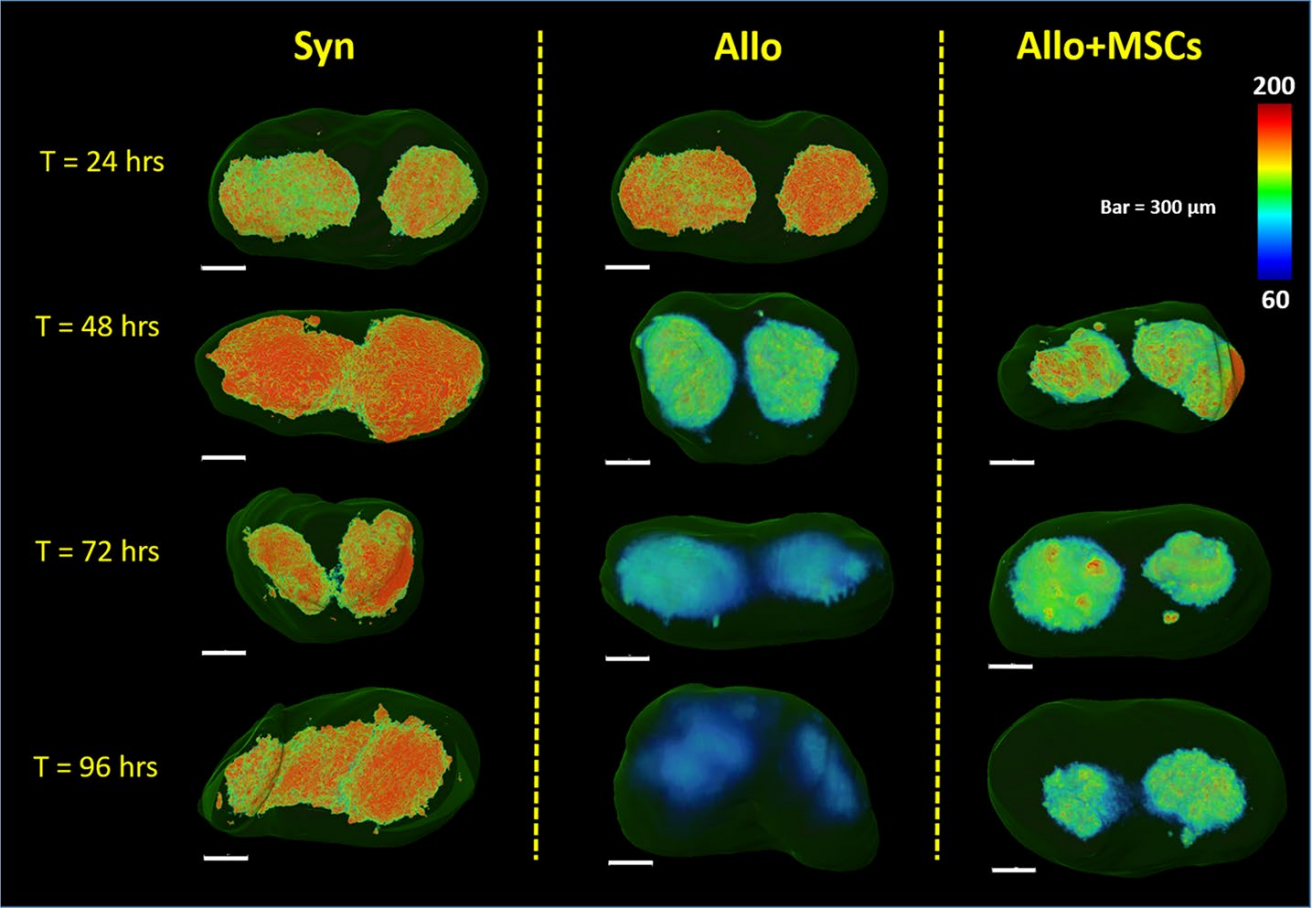
Volume rendering of CFSE signal in the inguinal lymph nodes shows that alloreactive T-cells lost their CFSE intensity over time as they proliferated (second column). With MSC treatment, this CFSE dilution was attenuated (third column). In the syngeneic control, T-cells in the lymph nodes maintained high CFSE intensity which indicated minimal proliferation.

Color-coded volume rendering of CFSE intensity shows that donor-derived, allogeneic, T-cells aggregated in the spleen at early time points (24 and 48 h), and substantially lost their fluorescent intensity over time as they proliferated (72 and 96 h) (Fig. 9). By contrast, the signal in syngeneic spleens remained relatively constant over time (Fig. 9 and Suppl. Fig. 3). With MSC treatment, the rate of reduction in CFSE signal in the spleens of allo mice was markedly reduced. Similarly, allogeneic T-cells in representative (inguinal) lymph nodes exhibited a significant reduction in CFSE intensity over time, whereas syngeneic T-cell intensity remained high (Fig. 10). Again, with MSC treatment, the rate of reduction of CFSE signal in allogeneic lymph nodes was reduced. These qualitative results suggest that CFSE dilution is a powerful tool for assessing T-cell proliferation in the animal model, and clearly illuminating immunomodulatory effects of MSCs occurring within the SLOs.

**Figure 11:**
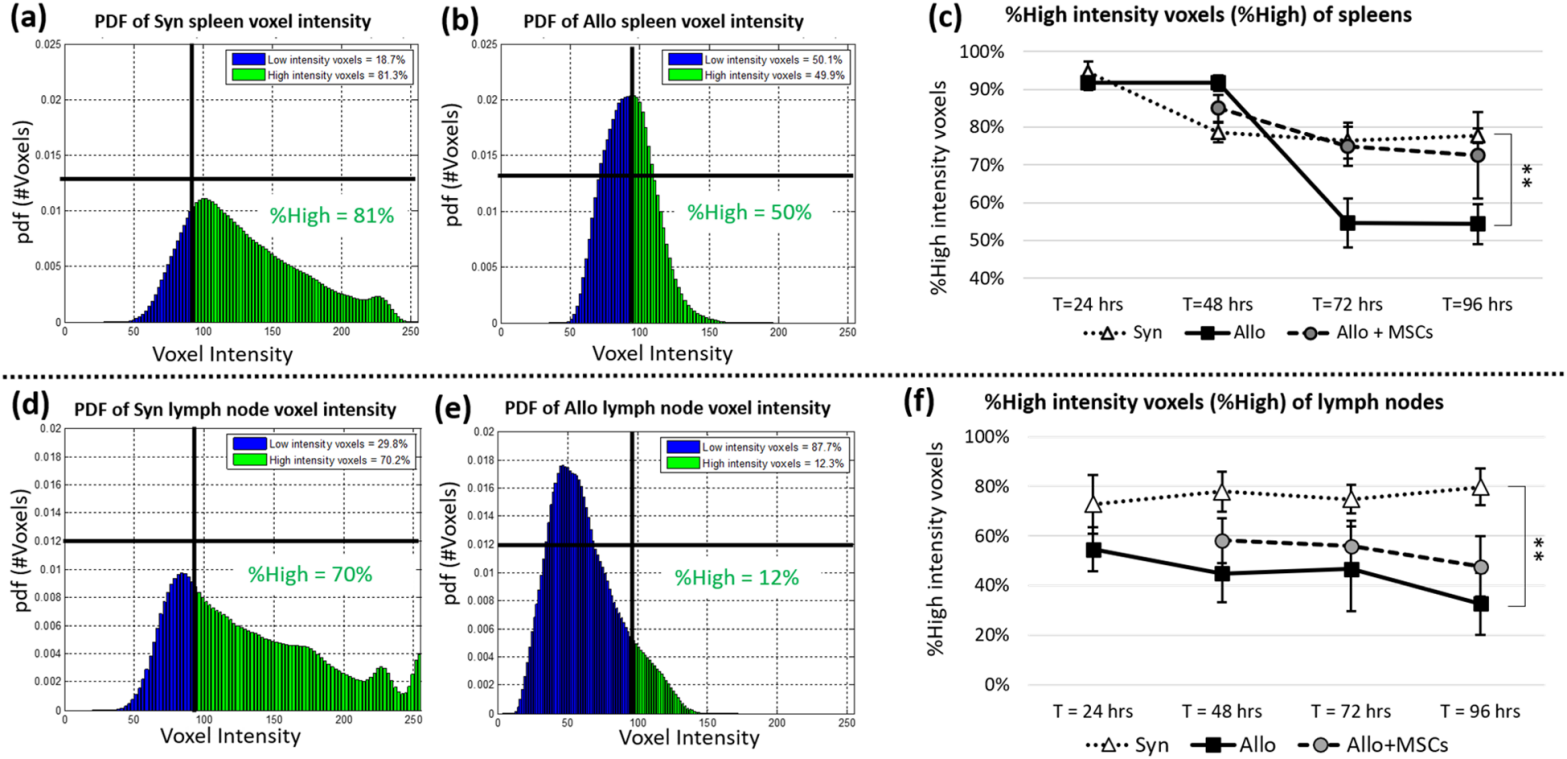
%High intensity voxels (%High) could be used to assess T-cell proliferation in SLOs. Voxels of the SLOs were used to estimate probability density function (PDF) of voxel intensity. The estimated PDFs of a Syn spleen and an Allo spleen are shown in (**a**) and (**b**), respectively (T = 72 h post BMT). Similar results from representative inguinal lymph nodes are shown in (**d**) and (**e**) (T = 72 h post BMT). %High was defined as the proportion of high intensity voxels (i.e. those with intensity greater than a threshold) to total voxels considered. This number could be visualized as an area under the curve (green area) of the probability density function. We argue that the %High is proportional to number of T-cells that maintained high concentration of CFSE. When we applied this assay to all study groups, results suggested that %High from the allogeneic group (solid line in (**c**) and (**f**)) was significantly lower than %High from the syngeneic group (dotted line in (**c**) and (**f**)) which suggests higher proliferation of T-cells in the allogeneic group. With MSC treatment, the CFSE dilution of alloreactive T-cells was greatly attenuated as compared to the untreated group (dashed line in (**c**) and (**f**)). Error bar represents standard error. ** represents significantly difference with *p*-value < 0.005 (Two-tailed Student’s t-test).

Probability density function (PDF) of CFSE voxel intensity and %High could also be used as quantitative metrics for assessing T-cell proliferation. Results (Fig. 11a,b) show that spleen PDF of syngeneic group was positively skewed while the spleen PDF of allogeneic group was less skewed and almost Gaussian-like in shape. This indicates that syngeneic T-cells in the representative spleen retained high concentration of the CFSE dye while allogeneic T-cells lost their dye concentration due to rapid cell division. By setting a reference threshold, we partitioned the area under PDF curve into high intensity voxels and low intensity voxels. %High was used to measure proportion of high intensity voxels to total detections. By applying this technique, we observed that %High of allogeneic spleens was significantly smaller as compared to the %High of syngeneic spleens by 72 h post BMT (Fig. 11c). The same observation was also true of the lymph nodes. We also observed positively skewed PDF and higher %High value in the syngeneic group (Fig. 11d), and less skewed PDF and lower %High value in the allogeneic group (Fig. 11e). Again, we observed %High of allogeneic lymph nodes significantly decreased over time as compared to %High of syngeneic lymph nodes (Fig. 11f). As expected, with MSC treatment, the rate of %High reduction (with respect to time) in both spleen and lymph nodes was greatly diminished (Fig. 11c,f).

## Discussion

Microscopic cryo-imaging enables tracking of fluorescently labeled T-cells and MSCs anywhere in a whole mouse with single cell sensitivity. Exogenous, donor derived, T-cells were mostly found in lung, liver, bone marrow, secondary lymphoid organs and the GI-tract while MSCs were mostly found in lung, liver, spleen, and bone marrow. Consistent with previous reports^35,42^, the cellular bio-distribution is not simply related to blood flow. Rather cells preferentially deposit in specific organs are likely driven in part by inflammation or immune dysregulation. In our experiments, exogenous T-cells were found to home to the white pulp of spleen and paracortex of lymph nodes, regions known to participate in T-cell priming and expansion^56^. MSCs were found to co-localize in these SLOs, specifically accumulating in the marginal zone of the spleen and in the vicinity of the paracortex in the lymph nodes.

We observed a significantly greater number of MSCs in spleens of mice in the allogeneic group compared to spleens of mice in the syngeneic group (Fig. 6a and Suppl. Table 1). This result is consistent with observations from other groups suggesting that MSCs can be primed and activated under systemic inflammatory conditions^26^. Interestingly, we found fewer numbers of MSCs in lymph nodes where the total numbers were comparable between the allogeneic and the syngeneic groups (Suppl. Table 2). It is possible that MSCs do not have lymph node homing receptors (such as CD62L and CCR7), which are required to get into lymph node parenchyma^57^. However, it is also possible that inguinal and cervical nodes play a less prominent role in T cell activation after BMT compared to mesenteric lymph nodes and Peyer’s patches, which have been shown to be the primary sites of T cell early activation during GVHD induction^32,58^. In either case, we were still able to observe MSC immunomodulatory effects in the lymph nodes in these more peripheral lymph node locations.

Our volumetric analysis proved to be a useful assay for assessment of in vivo T-cell proliferation in GVHD mice. Using the three metrics described in methods, our results show that spleens of mice in the allogeneic group were significantly larger than spleens collected from the syngeneic group by 72 h post BMT, indicative of higher T-cell proliferation in the allogeneic setting (Figs. 7 and Suppl. Fig. 1). Among the three proposed metrics, WP volume (mm^3^) and %WP were better indicators for assessing T-cell proliferation as nearly all the volume change was due to white pulp enlargement. Similarly, the measured volume of lymph nodes from the allogeneic group was significantly greater than the syngeneic controls (Figs. 8 and Suppl. Fig. 2). Although in this study we chose inguinal and cervical lymph nodes as representatives, we believe that this technique is applicable to other types of lymph nodes as well. In sum, the proposed volumetric assays help differentiate the allogeneic from the syngeneic BMT recipients and proved to be an important tool for assessment of in vivo T-cell proliferation for the GVHD mouse model.

CFSE dilution proved to be an equally powerful assay for analyzing T-cell proliferation. T-cell proliferation was assessed by the reduction of CFSE intensity in the SLOs. The proposed methods included: (1) Color-coded volume rendering as a qualitative method, and (2) %High intensity voxels as a quantitative method. Our results from the splenic analysis showed that the CSFE signal in allogeneic BMT recipients rapidly increased at earlier time points (24 and 48 h) but then diminished at later time points (72 and 96 h). By contrast, the CFSE signal in the spleens of syngeneic animals was relatively consistent across all time points. We believe that the early aggregation of allogeneic T-cells in spleen is suggestive of their activation under during GVHD induction, and loss of CFSE signal over time is consistent with rapid proliferation. Our results were consistent with CFSE-based lymphocyte proliferation assays previously reported in the literature^52–54,59^. We repeated our analyses on peripheral lymph nodes, and results were consistent with those obtained in the spleens (Figs. 10 and 11f).

As noted in the introduction, GVHD is fundamentally dependent on interactions between donor T cells and host antigen presenting cells primarily, but not exclusively in secondary lymphoid organs^1,60,61^. We and others have uncovered key contributions of regenerative stomal cells (both mesenchymal stem cells (MSC) and multipotent adult progenitor cells (MAPC)) to these critical interactions and have specifically worked to define the biodistribution and mechanisms by which T cell activation and expansion are regulated regenerative stromal cells following allo-BMT^21,35,62,63^. Indeed, key metabolic pathways underlying MSC and MAPC-mediated immunomodulation^64^ have significant implications with respect to their use in the prevention and treatment of GVHD^28,29,65^. The goal of the laboratory-based studies described herein was to develop novel imaging techniques to both illuminate mechanisms by which stromal stem cells regulate inflammation engendered after allogenic BMT and inform the rational design of future clinical trials to optimize the use of these cells for GVHD prevention and or treatment in the clinic. Indeed, the use of stromal stem cells in the context of allogeneic BMT has shown promise in some, but not all clinical trials^66–68^.

In conclusion, we have shown that multispectral cryo-imaging can be used for assessing immunomodulatory effects of MSCs on experimental GVHD as well as for tracking the 3D biodistribution of the cells of interest anywhere in the mouse. Homing sites of donor-derived T-cells and injected MSCs were identified addressing in part an unmet need associated with clinical trials using MSCs for GVHD^66–69^. In addition, MSCs modulate T-cell activation in vivo; SLO enlargement and CFSE dilution assays demonstrated that T-cell proliferation was greatly attenuated in the MSC treated group. This can be observed visually (Figs. 9 and 10) and quantitatively (Fig. 11). Our observations were consistent in both the spleen and lymph nodes. Notably, MSCs did not completely suppress T-cell proliferation to the level observed in the syngeneic animals, an effect we believe is critical for anti-infective immunity and maintaining graft-versus-leukemia effects^70^. In aggregate, our findings support that notion that MSCs may represent a novel strategy to prevent or treat GVHD as has been suggested in recent clinical trials^28,29,66–69^. Importantly, our methods are not limited only to characterizing immune dysregulation during GVHD; they represent useful tools to study cell division and biodistribution in similar models of immunological diseases such as infection, autoimmune disease and cancer. In this context, our imaging techniques represent promising alternatives to traditional lymphocyte proliferation assays.

## Supplementary Information

Supplementary Information 1.

Supplementary Video 1.

Supplementary Video 2.

Supplementary Video 3.

Supplementary Video 4.

## Data Availability

The datasets generated during and/or analyzed during the current study are available from the corresponding author on reasonable request.
