# Author Correction: Assessment of the therapeutic role of mesenchymal stromal cells in a mouse model of graft-versus-host disease using cryo-imaging

**DOI:** 10.1038/s41598-023-30113-0

**Published:** 2023-02-21

**Authors:** Patiwet Wuttisarnwattana, Saada Eid, David L. Wilson, Kenneth R. Cooke

**Affiliations:** 1 Optimization Theory and Applications for Engineering Systems Research Group, Department of Computer Engineering, Excellence Center in Infrastructure Technology and Transportation Engineering, Biomedical Engineering Institute, Chiang Mai University, Chiang Mai, Thailand; 2 Department of Pediatrics, Case Western Reserve University, Cleveland, OH USA; 3 Department of Biomedical Engineering, Case Western Reserve University, Cleveland, OH USA; 4 Department of Oncology, The Sidney Kimmel Comprehensive Cancer Center at Johns Hopkins Hospital, Johns Hopkins University, Baltimore, MD USA

Correction to: *Scientific Reports* 10.1038/s41598-023-28478-3, published online 30 January 2023

The original version of this Article contained errors. The title of this paper

“Assessment of therapeutic role of mesenchymal stromal cells in mouse models of graft-versus-host disease using cryo-imaging”

now reads:

“Assessment of the therapeutic role of mesenchymal stromal cells in a mouse model of graft-versus-host disease using cryo-imaging”

In addition, References 40, 41, 46 and 49 contained errors, where the titles and the DOI of the respective works were omitted.

The correct references are listed below:

**Reference 40:**

Ketson, P. & Wuttisarnwattana, P. White Pulp Segmentation Algorithm for Mouse Spleen Cryo-imaging Data Using U-Net. *Proceedings of the 2020 4th International Conference on Vision, Image and Signal Processing (ICVISP),* **Article 6**, 1–7**.** https://doi.org/10.1145/3448823.3448834 (2020).

**Reference 41:**

Wuttisarnwattana, P. Automatic whole mouse segmentation for cryo-imaging data using DRLSE model. *Proceedings of the 2016 13th International Conference on Electrical Engineering/Electronics, Computer, Telecommunications and Information Technology (ECTI-CON),* 1-5. https://doi.org/10.1109/ECTICon.2016.7561436 (2016).

**Reference 46:**

Chatboonward, T. & Wuttisarnwattana, P. Biliary Tract Autofluorescence Cleaning for Liver Cryo-imaging Data. *Proceedings of the 2021 18th International Conference on Electrical Engineering/Electronics, Computer, Telecommunications and Information Technology (ECTI-CON),* 650-653. https://doi.org/10.1109/ECTI-CON51831.2021.9454766 (2021).

**Reference 49:**

Wuttisarnwattana, P., Raza, S., Eid, S., Cooke, K. & Wilson, D. Novel T Lymphocyte Proliferation Assessment Using Whole Mouse Cryo-Imaging. *Proceedings of the 2014 SPIE Medical Imaging: Biomedical Applications in Molecular, Structural, and Functional Imaging (SPIE MI),* **9038**, https://doi.org/10.1117/12.2042960 (2014).

The original Article and accompanying Supplementary Information 1 file have been corrected.

