# Supplementary material for: Assessment of the therapeutic role of mesenchymal stromal cells in a mouse model of graft-versus-host disease using cryo-imaging
Source: Sci Rep. 2023 Jan 30;13:1698. doi: 10.1038/s41598-023-28478-3 (PMC9886911; doi:10.1038/s41598-023-28478-3)
Supplement: Supplementary file 1 — Supplementary Information 1. [file 41598_2023_28478_MOESM1_ESM.docx]

Assessment of the Therapeutic Role of Mesenchymal Stromal Cells in a Mouse Model of Graft-Versus-Host Disease using Cryo-imaging:

Supplemental Data

Patiwet Wuttisarnwattana^a, *^, Saada Eid^b^, David L. Wilson^c^, and Kenneth R. Cooke^d^

^a^Optimization Theory and Applications for Engineering Systems Research Group, Department of Computer Engineering, Excellence Center in Infrastructure Technology and Transportation Engineering, Biomedical Engineering Institute, Chiang Mai University, Chiang Mai, Thailand;

^b^Department of Pediatrics, Case Western Reserve University, Cleveland, OH, USA;

^c^Department of Biomedical Engineering, Case Western Reserve University, Cleveland, OH, USA

^d^Department of Oncology, The Sidney Kimmel Comprehensive Cancer Center at Johns Hopkins Hospital, Baltimore, MD, USA;

**Supplemental Table 1.** Numbers of detected MSCs in spleens.

| Time points  (hours post BMT) | Syngeneic group  (cells) | Allogeneic group  (cells) | p < 0.05 |
| --- | --- | --- | --- |
| T=48 | 2,318 ± 847 | 4,491 ± 795 |  |
| T=72 | 1,653 ± 247 | 3,460 ± 252 | * |
| T=96 | 1,120 ± 113 | 2,830 ± 744 | * |
| Numbers represent mean ± standard error | | | |

**Supplemental Table 2.** Numbers of detected MSCs in lymph nodes.

| Time points  (hours post BMT) | Inguinal lymph nodes (cells) | | Cervical lymph nodes (cells) | |
| --- | --- | --- | --- | --- |
|  | Syn | Allo | Syn | Allo |
| T=72 | 87 ± 5 | 112 ± 13 | 83 ± 13 | 91 ± 16 |
| T=96 | 103 ± 10 | 84 ± 17 | 79 ± 16 | 62 ± 9 |
| Numbers represent mean ± standard error | | | | |

**Additional description regarding culture and expansion of human bone marrow-derived mesenchymal stromal cells (hMSCs) used in this study**

Bone marrow (BM) aspirates from five healthy donors were used to produce human MSCs (hMSCs) used in this study [1]. Written informed consents are obtained from all patients for the procedure in line with the University Hospitals Case Medical Center's Institutional Review Board (UHCMC IRB protocol 09-90-195). The research was performed in accordance with the Declaration of Helsinki. Technicians at the Hematopoietic Stem Cell Facility at the Case Comprehensive Cancer Center gathered and prepared specimens. BM aspiration (10–30 ml) on adult volunteer donors was performed under local anesthetic. Mononuclear cells were obtained by Percoll gradient centrifugation (1.073 gm/ml) and plated at a density of 1.7 x 10^5^ cells/cm^2^ in 175 cm^2^ tissue culture flasks in complete hMSC medium (DMEM low glucose, supplemented with 1% antibiotic/antimycotic, and 10% fetal bovine serum from selected lots; all reagents from Gibco-Invitrogen, Carlsbad, CA.) The media was changed every three to four days and non-adherent cells were removed after the cells had been given 72 hours to adhere. At a density of 2–6 x 10^3^ cells/cm^2^ per 175 cm^2^ (referred to as "passage"), adherent cells were subcultured by trypsinization once cultures reached 80–90% confluency. Our experiment utilized third- to fifth-passage of the hMSCs. The hMSC phenotype was verified by morphology, flow cytometry (CD45^-^CD105^+^CD90^+^CD80^-^CD73^+^HLA-I^+^), and in vitro differentiation into osteoblasts, chondroblasts, and adipocytes (Dominici 2006).

[1] Auletta JJ, Zale EA, Welter JF, et al. Fibroblast Growth Factor-2 Enhances Expansion of Human Bone Marrow-Derived Mesenchymal Stromal Cells without Diminishing Their Immunosuppressive Potential. Stem Cells Int. 2011;2011:235176.

[2] Dominici M, Le Blanc K, Mueller I, et al. Minimal criteria for defining multipotent mesenchymal stromal cells. The International Society for Cellular Therapy position statement. Cytotherapy. 2006;8:315-317


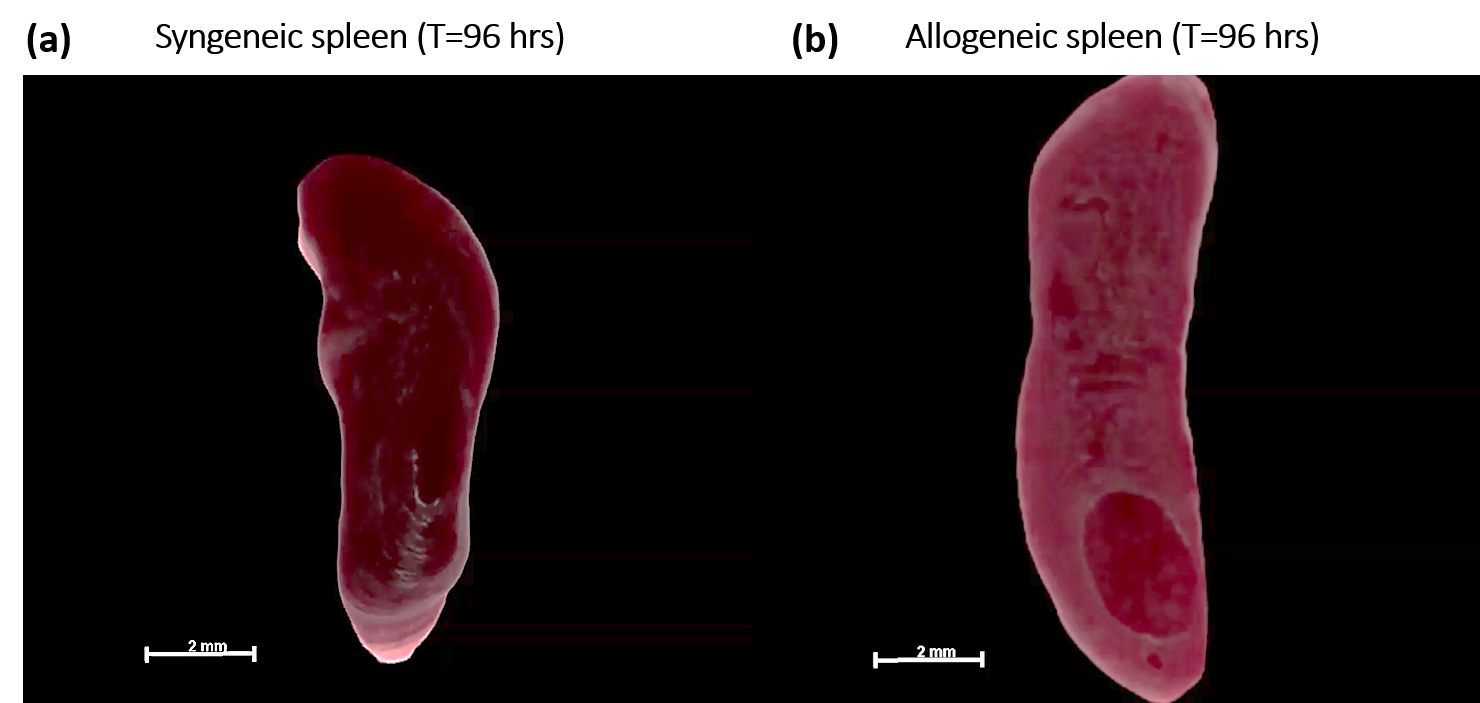


**Supplemental figure 1:** Spleen enlargement can be used to assess T-cell proliferation. Volume rendering of representative spleens show that the spleen of allogeneic mouse was bigger than the spleen of syngeneic mouse. Alloreactive T-cells and other immune cells proliferated rapidly resulted in increased cellularity of the spleen while syngeneic T-cells proliferated minimally or not at all. (Bar = 2 mm)


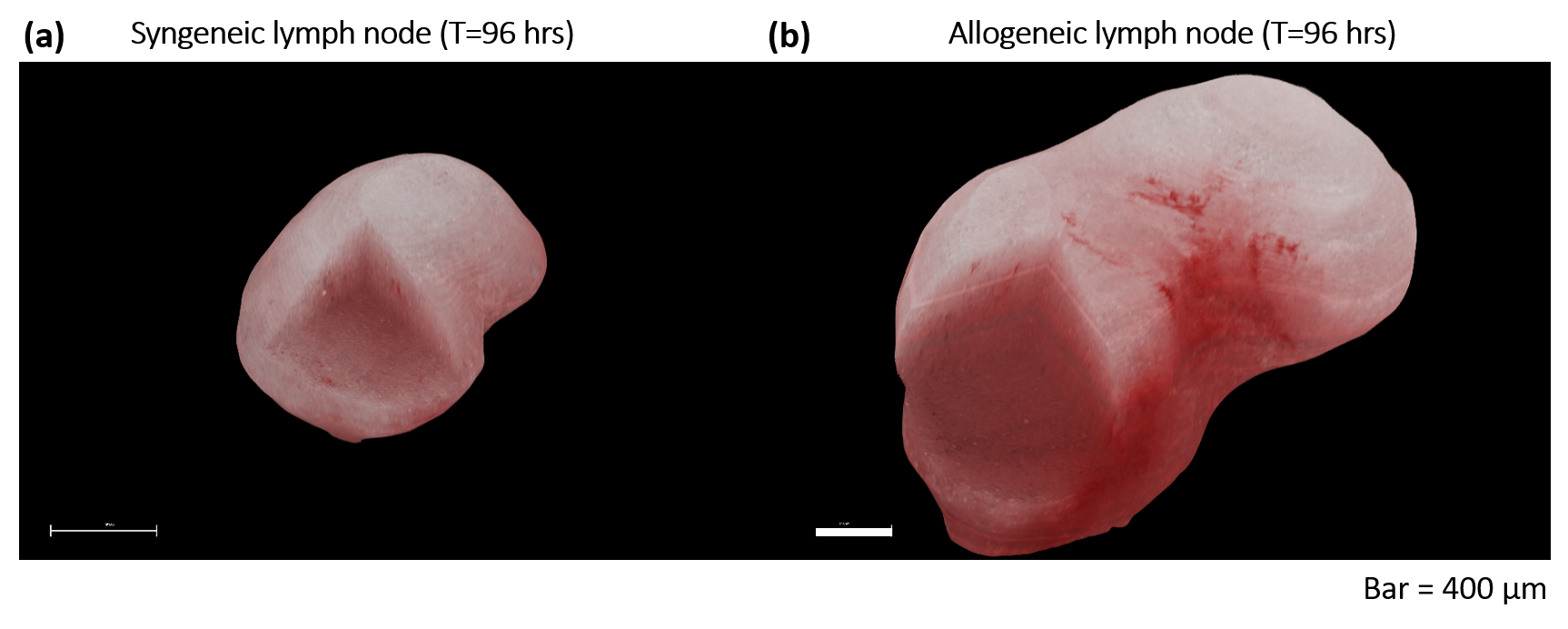


**Supplemental figure 2:** Lymph node enlargement can be used to assess T-cell proliferation. Volume rendering of representative (inguinal) lymph nodes show that lymph node of allogeneic mouse was significantly bigger than the lymph node of the syngeneic control. Alloreactive T-cells and other immune cells proliferated rapidly resulted in increased cellularity in the lymph node while syngeneic T-cells proliferated minimally or not at all. (Bar = 400 µm)


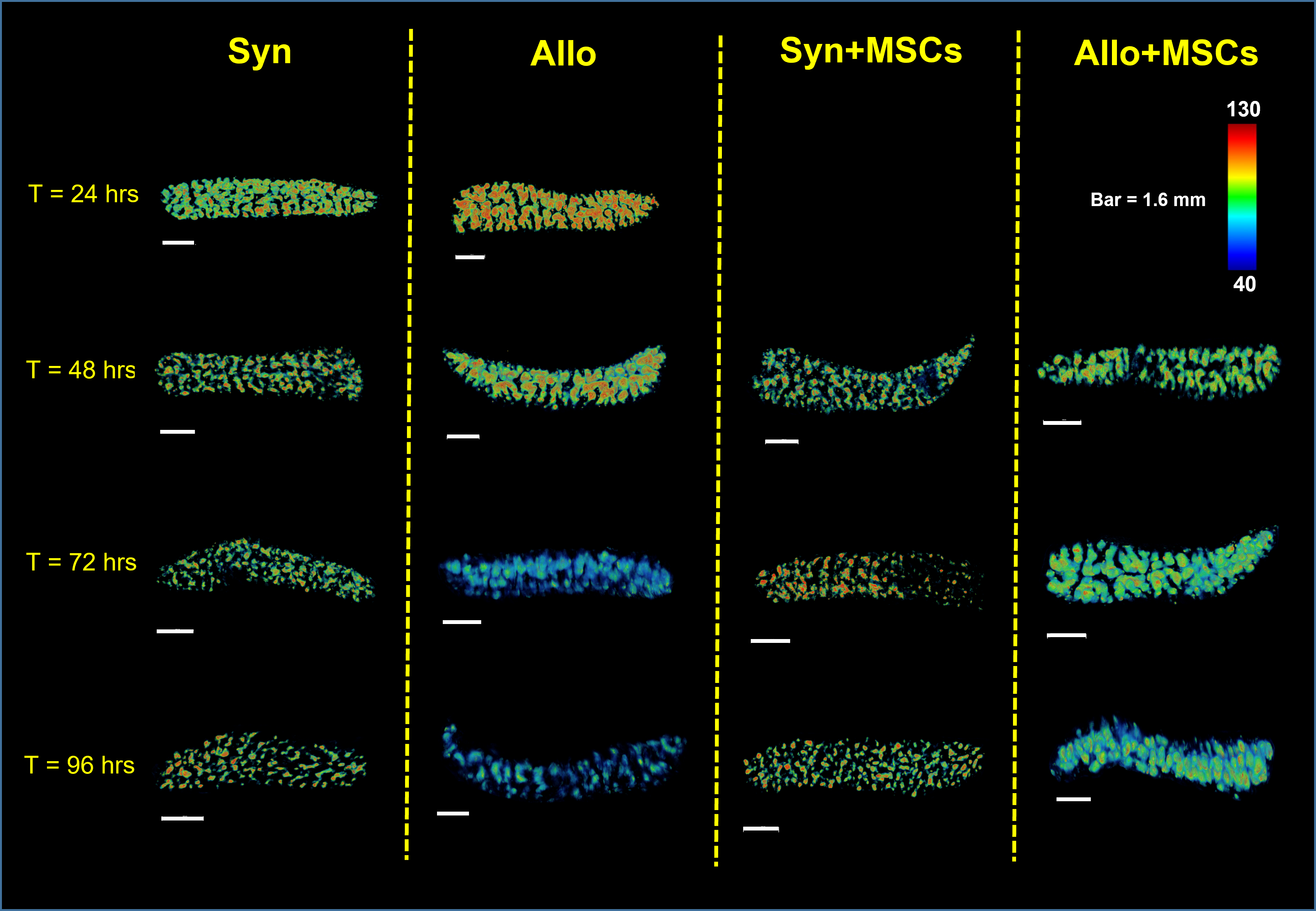


**Supplemental figure 3:** CFSE dilution assays with additional Syn+MSCs group. There was no CFSE dilution effect observed in the syn group as the cells minimally proliferated. This visualization shows that all syn groups were similar with or without MSC treatment. This observation holds true in other secondary lymphoid organs such as lymph nodes (data not shown).


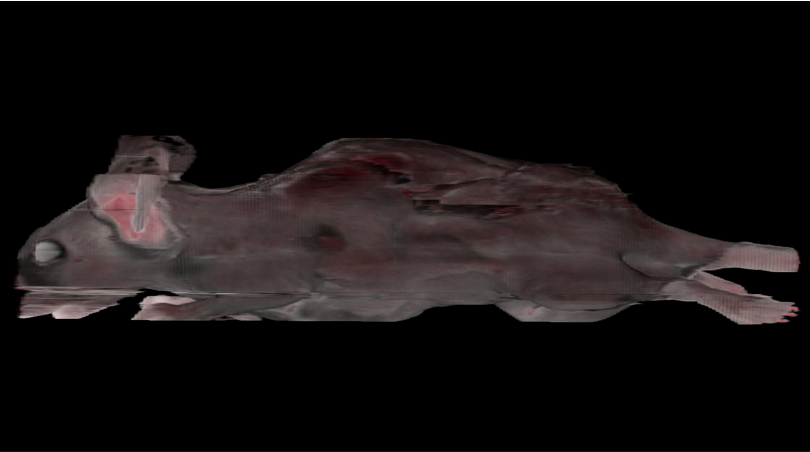


**Supplemental media 1:** T-cell and MSC bio-distribution in a mouse

<<This is a screen capture of the video, please see the video on the publisher website>>


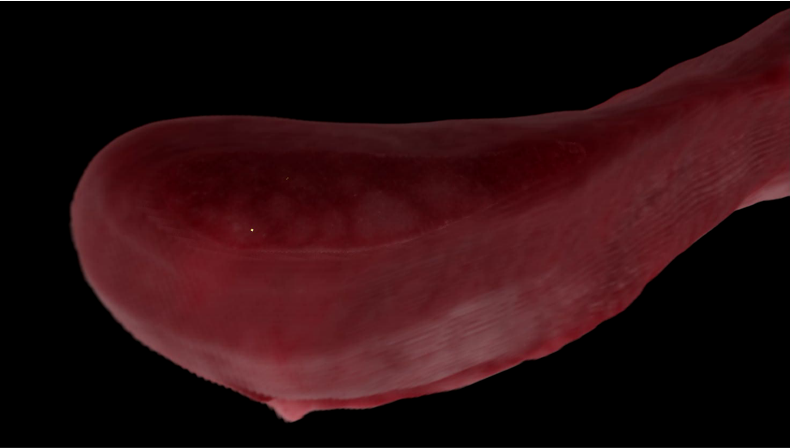


**Supplemental media 2:** T-cell and MSC bio-distribution in a mouse

<<This is a screen capture of the video, please see the video on the publisher website>>


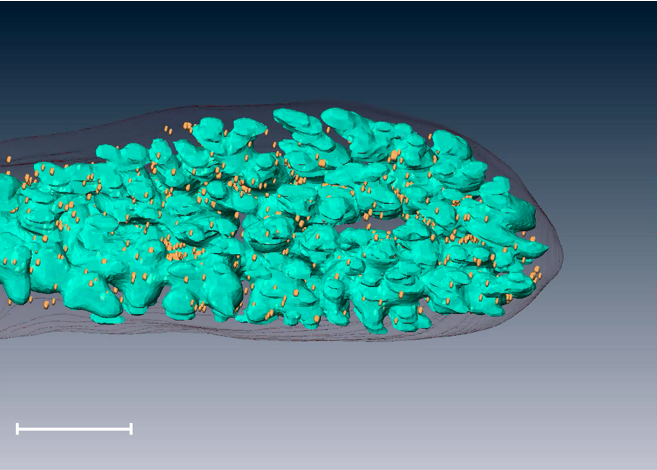


**Supplemental media 3:** Surface rendering showing hMSCs homed to the marginal zone of the spleen (bar = 1.2 mm)

<<This is a screen capture of the video, please see the video on the publisher website>>


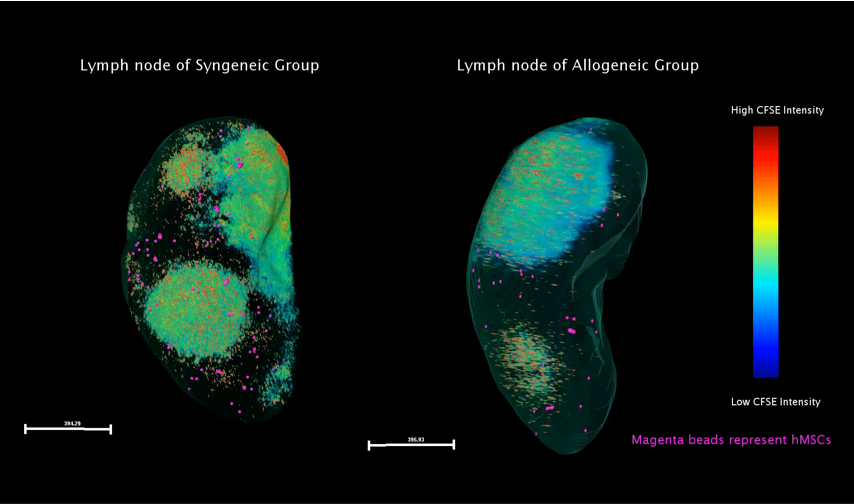


**Supplemental media 4:** Animation of 3D renderings from representative (inguinal) lymph nodes show that hMSCs were found in lymph nodes. CFSE-labeled T-cells were color-coded according to their intensity. (Bar = 395 µm)

<<This is a screen capture of the video, please see the video on the publisher website>>
